# Association of Sensory Liking for Fat with Dietary Intake and Metabolic Syndrome in Korean Adults

**DOI:** 10.3390/nu10070877

**Published:** 2018-07-06

**Authors:** Hyeyoung Park, Yoonjin Shin, Oran Kwon, Yangha Kim

**Affiliations:** Department of Nutritional Science and Food Management, Ewha Womans University, Seoul 03760, Korea; hypark1597@naver.com (H.P.); yjin19@hotmail.com (Y.S.); orank@ewha.ac.kr (O.K.)

**Keywords:** fat liking, metabolic syndrome, nutrient intake, obesity

## Abstract

Individual sensory liking is perceived as a major determinant of dietary intake and may influence chronic disease. This study aimed to assess the odds of metabolic syndrome in Korean adults based on their liking for fat. Data from 7731 adults aged 40–69, included in the Korean Genome and Epidemiology Study, were collected. Fat liking scores were obtained from self-report questionnaires. In both genders, sensory liking for fat was positively associated with consumption of red meat and added fat. Subjects with a stronger liking for fat showed a higher intake of energy and fat and a lower intake of vitamin C and fiber as compared to subjects with a lower liking for fat. There were increasing trends in the odds of metabolic syndrome with stronger liking for fat (odds ratios (ORs) for the Like group compared to the Dislike group, men: ORs = 1.29 (95% confidence interval 1.06–1.50) *p*-trend = 0.01; women: ORs = 1.28 (1.04–1.58) *p*-trend = 0.018) after adjustment for age, alcohol intake, smoking, exercise, education level, and income status. Our results suggested that the liking for fat-rich food might partially contribute to the increased odds of metabolic syndrome.

## 1. Introduction

Metabolic syndrome is a common disorder associated with increased risk of cardiovascular disease and type 2 diabetes [1]. The predominant risk factors for metabolic syndrome are abdominal obesity and insulin resistance, and other potentially related conditions are physical inactivity, cigarette smoking, and poor diet [2,3,4]. Although multiple factors influence metabolic syndrome, the syndrome appears to be comparatively rare when there is no excess body fat [2]. As obesity increases, the prevalence of metabolic syndrome also increases [2]. Obesity thus can be said to be the major driver of the syndrome. Lifestyle changes could reverse metabolic risk, and dietary habits are considered an important factor affecting the prevention of metabolic syndrome.

Fat is universally preferred since it can provide a palatable flavor and a soft and crisp texture when mixed with other ingredients. Liking for fat often leads to the consumption of high-calorie foods, such as butter, meat, and fatty–sweet products [5,6]. Fatty foods give a sense of pleasure to eating due to the sensory properties they drive, which may encourage their overconsumption [7]. Excessive intake of energy has been reported to be closely associated with major chronic disease, including obesity, cardiovascular disease, and some cancers [8,9].

Previous studies reported a positive association between fat liking and body mass index (BMI) [10,11]. Moreover, the study conducted in French population found that adults who liked fat had a significantly increased risk of becoming obese [12]. There was also a positive association between liking for fat and cardiovascular risk factors such as blood pressure [13]. Liking for fat is supposed to increase the consumption of high-fat and energy-dense foods and is highly likely to cause obesity-related metabolic disorders. However, the relationship between liking for fat and metabolic syndrome has yet to be investigated. Thus, the purpose of this study was to investigate the association of fat liking with dietary intake and metabolic syndrome in Korean adults.

## 2. Materials and Methods

### 2.1. Subjects

With the objective of identifying risk factors for chronic disease among Koreans, the Korean Genomic Epidemiology Study (KoGES) implemented health examinations for adult residents aged 40 to 69 years old in the Ansung and Ansan areas, thus representing rural and urban communities, and survey questionnaires were collected [14]. This study used KoGES data surveyed from May 2001 to February 2003. Among the 8840 people (4182 men and 4658 women), those who did not have data related to liking of fat (*n* = 117), body measurements (*n* = 12), blood tests (*n* = 257) or sociodemographic information (*n* = 362) were excluded. Those with a total daily calorie intake that was too high or too low (<800 or >4000 kcal for men, <500 or >3500 kcal for women) were also excluded [15] (*n* = 361). These exclusions left 7731 subjects (3750 men and 3981 women) who were appropriate for the final analysis. This study was approved by the Institutional Review Board of Ewha Womans University (85-4, December 2014), and the procedures followed were in accordance with the Helsinki Declaration of 1975, as revised in 2008.

### 2.2. Assessment of Liking for Fat

Dietary intake was collected by well-trained researchers using a semi-quantitative food frequency questionnaire (SQFFQ). The validation of the SQFFQ was reported previously [16,17]. Dietary data for designing SQFFQ were obtained from the Korea Health and Nutrition Examination Survey (KNHANES) in 1998. Two hundred and forty-nine foods, which were selected based on their 0.9 cumulative percent contribution, and 254 items, which were selected based on their 0.9 cumulative multiple regression coefficients, respectively, were categorized into 97 food groups based on their nutrient composition. Because of the seasonality of the survey, several popular Korean foods missing from the list were included. Subjects indicated the average intake frequency of 103 food items over a period of 12 months (on a 9-point scale of ‘almost none’, ‘once a month’, ‘twice or three times a month’, ‘once or twice a week’, ‘twice or three times a week’, ‘five or six times a week’, ‘once a day’, ‘twice a day’, and ‘three times a day’) and the average intake quantity (on a 3-point scale of small, medium, and large). The researchers presented the pictures of different quantities to the subjects for reference. Favorite cooking method was obtained by asking the subjects how often they had fried food, stir-fried food, seasoned food, and soups and stews (on a 5-point scale of ‘almost none’, ‘once–twice a month’, ‘once–three times a week’, ‘four–six times a week’, and ‘every day’). Then, the results were linearly transformed into values ranging from 0 to 10, with a high score indicating higher preference. Foods were selected from the list of SQFFQ and were classified into 16 food groups according to previous articles, with slight modification: meat (red meat, processed meat, and chicken), eggs, dairy products, fish and shellfish, seaweeds, grains, potatoes, beans, nuts, fruits, vegetables, mushrooms, added fat, cakes/cookies and chocolates, soft drinks, and other beverages [16]. The food intake determined through the SQFFQ was converted into average intake data for 23 nutrients using the Korean food composition and nutrition table [18]. The nutrient intake obtained by SQFFQ was slightly lower in protein and fat than in dietary records, but total energy and carbohydrate intake were not significantly different [17]. Fat liking was obtained by asking whether the subjects liked fat-rich food [10,19]. The possible answers to this question were ‘dislike it very much’, ‘dislike’, ‘neither dislike nor like’, ‘like’, and ‘like it very much’. Subjects were classified into three groups according to their level of liking for fat: Dislike (dislike it very much and dislike), Neither like nor dislike, and Like (like and like it very much).

### 2.3. Classification of Obesity and Metabolic Syndrome

According to the World Health Organization’s Asia-Pacific Area criterion [20], obesity is indicated by a BMI greater than or equal to 25.0 kg/m^2^. The diagnosis of metabolic syndrome was performed based on the diagnostic criteria presented in the 2009 Joint Scientific Statement [21]. Individuals that meet more than three of the following conditions are diagnosed with metabolic syndrome: waist circumference: ≥90 cm for men and ≥80 cm for women according to the recommendation by International Diabetes Federation [3], high-density lipoprotein (HDL) cholesterol <40 mg/dL for men and <50 mg/dL for women or treatment of dyslipidemia, triglycerides ≥150 mg/dL or treatment of dyslipidemia, systolic and diastolic blood pressure ≥130 and 85 mmHg or antihypertensive treatment, fasting glucose ≥100 mg/dL or treatment of type 2 diabetes.

### 2.4. General Characteristics, Anthropometric Measurements, and Biochemical Variables

General information on gender, age, alcohol consumption (g/day), current smoking (yes, no), moderate-intensity physical activity (yes, no), education status (<high school, ≥high school), and monthly income (<2 million Korean won (KRW), ≥2 million KRW) was collected using an interview administered questionnaire.

The height and weight of the subjects were measured under the condition that each subject took off his or her shoes and clothes and wore only a patient gown. BMI was calculated as weight (kg)/height (cm^2^). The circumferences of the waist and hip were measured by non-stretchable standard tape while each subject stood with their legs together and both arms stretched laterally. The circumference of the waist was calculated by measuring the thinnest part of the waist between the ribs and long bone, and the circumference of the hip was estimated by measuring the protruding part of the hip. Each measurement was performed three times to calculate the average values. The waist and hip circumference ratio was also calculated by dividing the waist average value by the hip average value. Blood pressure was measured three times at intervals of 30 safter 5 min of rest, and the average values were calculated and used for the study.

All subjects had their blood samples taken after having fasted for more than 8 hours. The collected blood samples were centrifuged at the site and then analyzed for total cholesterol, HDL cholesterol, triglyceride, hemoglobin A1c (HbA1c), fasting glucose, and fasting insulin. The homeostasis model assessment of insulin resistance (HOMA-IR) was calculated as fasting insulin (μIU/mL) × fasting glucose (mmol/L)/22.5 [22]. Low-density lipoprotein (LDL) cholesterol was calculated by the following equations described by Friedewald with triglyceride concentrations <400 mg/dL [23]: LDL cholesterol = [Total cholesterol (mg/dL) − {HDL cholesterol (mg/dL) − (Triglycerides (mg/dL)/5)}].

### 2.5. Statistical Analysis

Analyses were performed separately for men and women, since sex interactions with anthropometric, economic, and lifestyle variables were found. The continuous variables are represented as the mean and standard error (SE) to evaluate the observed variation around the calculated regression line [24]. The trend *p* was obtained through general linear model analysis and the Cochran–Mantel–Haenszel analysis. The general linear model and the Cochran–Mantel–Haenszel analysis with adjustment for age were used to determine differences in means and distribution of general characteristics and to test for linear trends according to level of liking for fat. Variables that are statistically significant in univariate analysis or known to be potentially important factors related to fat liking were considered as potential confounders, such as age, alcohol intake, smoking, exercise, education level, and income status were adjusted in analyses. For the association between liking for fat and odds of obesity and metabolic syndrome, logistic regression analysis was carried out. All data were analyzed using SAS v9.4 (SAS Institute Inc., Cary, NC, USA) with the significance level set at *p* < 0.05.

## 3. Results

The general characteristics of the subjects are summarized in Table 1. The number of subjects in the Like group was 857 (22.9%) for men and 528 (13.3%) for women. Compared with subjects with a lower liking for fat, men and women with a stronger liking for fat tended to have higher weight. Men with a stronger liking for fat were more likely to drink alcohol and exercise. Women with a stronger liking for fat tended to be younger. In addition, there were significant positive associations between liking for fat and preference fried/stir fried food in men and women.

Food consumption according to level of liking for fat is shown in Table 2. Men and women with a stronger liking for fat tended to have higher intakes of red meat, added fat, and soft drinks, and lower intake of vegetables after adjustment for age, BMI, alcohol intake, smoking, exercise, education level, and income status.

Daily nutritional intake classified by level of liking for fat is shown in Table 3. Men and women with a stronger liking for fat showed significantly higher energy, fat, and cholesterol intake and lower carbohydrate, vitamin C, and fiber intake after adjustment for age, BMI, alcohol intake, smoking, exercise, education level, and income status. They consumed more energy from fat but less from carbohydrates.

Clinical characteristics according to level of liking for fat are shown in Table 4. Compared with subjects with a lower liking for fat, men and women with a stronger liking for fat tended to have higher total cholesterol, LDL cholesterol, waist circumference, and BMI. Men with a stronger liking for fat had higher LDL cholesterol concentrations. Women with a stronger liking for fat had a higher insulin and HOMA-IR.

Table 5 shows the odds for obesity and metabolic syndrome according to level of liking for fat. The overall prevalence of obesity in this subjects was 1530 (40.8%) for men and 1790 (45.0%) for women. There was a significant positive association between liking for fat and the odds for obesity in both men and women after adjustment for age, alcohol intake, smoking, exercise, education level, and income status and it was similar to the unadjusted results (Table S1). The odds ratios (ORs) (95% confidence interval (CI)) comparing the Like versus Dislike groups for obesity were 1.87 (1.58–2.21) in men and 1.72 (1.42–2.08) in women. In addition, the overall prevalence of abdominal obesity and metabolic syndrome in this subjects was 822 (21.9%) and 760 (20.3%) for men and 2154 (54.1%) and 1212 (30.4%) for women, respectively. There was a significant positive association between liking for fat and the odds for abdominal obesity; the ORs (95% CI) for abdominal obesity were 1.91 (1.58–2.31) in men and 1.58 (1.29–1.94) in women. The liking for fat was significantly positively associated with the odds for metabolic syndrome in both men and women after adjustment. The ORs (95% CI) comparing the Like versus Dislike groups were 1.29 (1.06–1.50) in men and 1.28 (1.04–1.58) in women.

## 4. Discussion

In this study, the association between liking for fat and metabolic syndrome was analyzed using large-scale cohort data from Korean adults. Liking for fat was associated with daily intake of vegetables, meat, added fat, and soft drinks in men and women. Liking for fat was positively linked to dietary energy and fat and inversely linked to vitamin C and fiber intake. In addition, there was a significantly positive association between liking for fat and odds of obesity and metabolic syndrome.

Our study showed that liking for fat was positively associated with red meat, added fat, soft drinks and chocolate intake, and inversely associated with vegetable intake both in men and women. In addition, men and women with a stronger liking for fat tended to have higher energy and fat intake and lower fiber intake. Mejean et al. [5] found that subjects with a strong liking for fat showed higher intake of total energy, saturated fat, meat, butter, and sweetened cream desserts and lower intake of fiber, fruits, and vegetables. According to the studies of Drewnowski [25,26], a strong preference for fatty foods was associated with high fat and low fiber intake. Fat is a highly enriched source of energy, and food containing such highly concentrated energy can contribute to excessive calorie consumption. Furthermore, individuals who prefer fat may less likely to consume healthy foods, such as fruits and vegetables, because they find them less tasty. Hence, they may tend to replace healthy foods with energy-dense variants. Therefore, liking for fat is presumably related to excessive intake of energy and fat and low fiber intake. In addition, liking for fat showed a significantly positive association with moderate exercise in men. Fat liking can increase motivation to exercise for energy consumption. It was reported that exercise affects compensatory eating behavior [27]. Consistent with this result, subjects with stronger liking for fat were more likely to exercise.

In this study, liking for fat had a significantly positive association with odds for obesity in men and women. These findings are similar to a previous report [28] that French adults with higher BMIs preferred fat. In middle-aged Japanese subjects, the fat-rich and heavy taste preferences had a positive association with an increase in body weight [10]. The preference for palatable tastes such as fat could lead to excessive energy intake, which increases body weight. On the other hand, low-energy-density diets (e.g., high in dietary fiber) were related to lower body weights [29]. Diets high in fiber are associated with lower body weight and body fat, possibly because they promote satiation, decrease absorption of macronutrients, and alter the secretion of gut hormones [30]. Our results have shown that liking for fat was related to low fiber intake and high energy and fat intake. This dietary intake can have long-term consequences on the risk of developing obesity. If all of these factors are taken into consideration, it can be presumed that there is a positive association between liking for fat and the obesity.

Liking for fat also appeared to be significantly positively associated with the odds of metabolic syndrome and abdominal obesity in both men and women. Mendoza et al. [31] reported that a highly energy-dense diet was associated with increased abdominal obesity and prevalence of metabolic syndrome. Dietary factors, particularly energy and fat intake, are strongly related to body fat deposition. Excess body fat reduces insulin sensitivity, thereby increasing the prevalence of the metabolic diseases [32]. The presence of insulin resistance may cause the development of type 2 diabetes [33]. Insulin resistance tends to interfere with glucose disposal and predisposes one to hyperinsulinemia and elevated plasma glucose. However, there was a significant inverse association between liking for fat and the odds of high fasting glucose in men. Men with stronger liking for fat tend to have higher intake of fat, yet significantly lower intake of carbohydrates as compared to subjects with lower liking for fat. Although there is conflicting evidence on the influence of carbohydrate intake on insulin sensitivity [34], a previous intervention study reported that after 6 months on a low-carbohydrate and high-fat diet, insulin sensitivity improved among obese subjects [35]. It is speculated that decreased carbohydrate intake in subjects with stronger liking for fat may have partially contributed to low odds of high fasting glucose.

Meanwhile, in metabolic syndrome patients, the numbers of individuals in the Dislike, Neither like nor dislike, and Like groups were 357 (47.0%), 204 (26.8%), and 199 (26.2%) for men, and 804 (66.3%), 232 (19.1%), and 176 (14.5%) for women, respectively. The proportion of the Like group with metabolic syndrome was expected to be higher than that of the Neither like nor dislike group (men: 658 (22.0%), women: 352 (12.7%)), but there was no significant difference. It is suggested that the liking for fat is not an independent predictor of metabolic syndrome, but rather that liking for fat and various other factors affect metabolic syndrome. Indeed, various influencing factors of the metabolic syndrome have been reported [36]. In the present study, a sensory liking for fat is proposed as one of the risk factors that affect health, with an investigation of the relationship between sensory fat liking and the risk of metabolic syndrome. There was a significant positive association between liking for fat and the risk of metabolic syndrome.

Sensory liking measures are intended to rapidly and accurately assess dietary behaviors [37]. According to basic research in cognition, recalling food likes/dislikes may be simpler and more accurate than recalling intake [38,39]. Because they require less cognition, sensory liking measures may be less biased by cognitive control than reporting dietary intake (i.e., dietary restraint). In this study, liking for fat was positively associated with fatty food consumption and risk of metabolic syndrome. These findings highlight the need to consider the influence of sensory liking in the management and prevention of metabolic disorders. Taking into account an individual’s liking may help dietitians and practitioners provide effective dietary counseling while supporting guidelines designed to reduce metabolic syndrome.

A liking for fat could influence the intake of both fatty–salty foods and fatty–sweetened foods [5]. Sensory properties of salt or sugar combined with fat may improve pleasure of meals and snacks and might therefore lead to higher consumption [40,41]. In addition, there is a report that the fat–salt sensation is strongly favored by heavy drinkers as compared to abstainers or irregular alcohol consumers [42]. Thus, creating an additional risk factor for obesity and health-related problems. Furthermore, genetic variation may also affect human fat sensation and consumption behavior. Sensitivity to the bitter taste has been shown to be genetically altered and positively associated with perceived intensity of fat and sugar, resulting in decreased liking for fatty or sweetened foods [43], whereas it has been associated with greater perceived intensity and liking for salt [44]. In a previous study, a representative fat receptor gene, the *CD36* gene polymorphism was reported to be associated with the perceived creaminess of salad dressings and the preference of certain types of fat in African Americans [45]. If such results are taken into consideration, it is postulated that various factors could modify the relationship between fat liking and metabolic disease.

There are some limitations in this study. As the relationship between fat liking and metabolic syndrome was analyzed through a cross-sectional study design, causality cannot be confirmed. In addition, dietary intake was analyzed through the SQFFQ and thus there may be an inaccurate quantification of actual intake amounts. However, despite such limitations, this is the first study to our knowledge to analyze the biochemical variables of subjects, such as total cholesterol, HDL cholesterol, triglyceride, and fasting glucose, and to identify associations between liking for fat and the metabolic syndrome in a large-scale population-based study in Korea.

## 5. Conclusions

This study of middle-aged Korean individuals demonstrated that liking for fat was positively associated with the odds of metabolic syndrome. In addition, subjects with a stronger liking for fat showed higher intake of energy and fat and lower intake of fiber. These findings suggested that the sensory liking for fat may be associated with the increased odds of metabolic syndrome in Korean adults. Although we believe that further longitudinal follow-up studies are needed, we expect that the findings of the study can be utilized as a guideline for diets designed to reduce metabolic disorders.

## Figures and Tables

**Table 1:** General characteristics of the participants according to the level of liking for fat.

	Men				Women			
	Dislike (*n* = 1895)	Neither Like nor Dislike (*n* = 998)	Like (*n* = 857)	*p*-Trend	Dislike (*n* = 2605)	Neither Like nor Dislike (*n* = 848)	Like (*n* = 528)	*p*-Trend
Age (years)	51.5 (0.2)	50.1 (0.3)	52.0 (0.3)	0.113	53.2 (0.2)	49.8 (0.3)	51.7 (0.4)	0.0003
Height (cm)	166.8 (0.1)	167.4 (0.2)	167.3 (0.2)	0.016	153.7 (0.1)	154.3 (0.2)	154.2 (0.2)	0.608
Weight (kg)	66.8 (0.2)	68.5 (0.3)	69.7 (0.3)	<0.0001	58.6 (0.2)	59.1 (0.3)	60.9 (0.4)	<0.0001
Alcohol intake (g/day)	51.5 (0.2)	50.1 (0.3)	52.0 (0.3)	0.016	53.2 (0.2)	49.8 (0.3)	51.7 (0.4)	0.608
Current smoker (%)	933 (49.2)	476 (47.7)	432 (50.4)	0.828	101 (3.9)	16 (1.9)	25 (4.7)	0.847
Moderate exercise (%)	936 (49.4)	539 (54.0)	458 (53.4)	0.026	1264 (48.5)	417 (49.2)	250 (47.4)	0.490
Education (≧high school, %)	1121 (59.2)	641 (64.2)	481 (56.1)	0.589	812 (31.2)	330 (38.9)	181 (34.3)	0.618
Monthly income (≧2 million KRW, %)	835 (44.0)	457 (45.8)	371 (43.3)	0.814	801 (30.8)	284 (33.5)	178 (33.7)	0.299
Favorite cooking method								
Fried food	2.3 (0.02)	5.0 (0)	7.6 (0.02)	<0.0001	2.2 (0.02)	5.0 (0)	7.6 (0.02)	<0.0001
Stir-fried food	3.9 (0.1)	4.1 (0.1)	4.4 (0.1)	<0.0001	4.1 (0.1)	4.4 (0.1)	4.8 (0.1)	<0.0001
Seasoned food	6.2 (0.1)	6.0 (0.1)	6.4 (0.1)	0.061	2.7 (0.1)	2.6 (0.1)	2.8 (0.1)	0.220
Soups and stews	9.3 (0.04)	9.2 (0.1)	9.3 (0.1)	0.919	1.8 (0.03)	2.1 (0.1)	1.9 (0.1)	0.226

KRW, Korean won. Values are expressed as means (SE) or number (percentage). The *p*-trend was obtained in general linear model analysis and Cochran–Mantel–Haenszel analysis with adjustment for age.

**Table 2:** Food consumption of the participants according to the level of liking for fat.

	Men				Women			
	Dislike (*n* = 1895)	Neither Like nor Dislike (*n* = 998)	Like (*n* = 857)	*p*-Trend	Dislike (*n* = 2605)	Neither Like nor Dislike (*n* = 848)	Like (*n* = 528)	*p*-Trend
Meat (g)	28.4 (0.5)	33.5 (0.7)	37.1 (0.8)	<0.0001	20.9 (0.4)	25.5 (0.7)	26.0 (0.9)	<0.0001
Red meat (g)	24.2 (0.4)	28.8 (0.6)	31.9 (0.7)	<0.0001	17.4 (0.3)	21.0 (0.6)	20.8 (0.8)	0.001
Processed meat (g)	0.4 (0.03)	0.6 (0.1)	0.7 (0.1)	<0.0001	0.4 (0.03)	0.7 (0.1)	0.7 (0.1)	0.0004
Chicken (g)	3.7 (0.1)	4.1 (0.1)	4.5 (0.2)	<0.0001	3.1 (0.1)	3.9 (0.2)	4.5 (0.3)	<0.0001
Eggs (g)	6.2 (0.2)	6.0 (0.2)	6.8 (0.3)	0.034	6.1 (0.2)	6.7 (0.3)	7.7 (0.4)	0.0002
Dairy products (g)	51.0 (1.3)	49.7 (1.7)	50.7 (2.0)	0.673	65.2 (1.4)	62.3 (2.4)	60.7 (2.8)	0.208
Added fat (g)	0.02 (0.004)	0.03 (0.005)	0.04 (0.006)	0.005	0.03 (0.003)	0.04 (0.01)	0.05 (0.01)	0.034
Fish and shellfish (g)	22.2 (0.4)	21.3 (0.5)	22.3 (0.6)	0.799	20.2 (0.3)	20.6 (0.6)	19.2 (0.6)	0.027
Seaweeds (g)	0.88 (0.02)	0.83 (0.02)	0.82 (0.02)	0.019	1.1 (0.02)	1.1 (0.04)	1.0 (0.04)	0.024
Grains (g)	411.9 (1.8)	406.3 (2.6)	394.3 (2.6)	<0.0001	410.6 (1.7)	400.9 (3.2)	398.0 (3.7)	0.024
Potatoes (g)	8.2 (0.2)	7.6 (0.3)	7.7 (0.3)	0.095	12.4 (0.3)	11.9 (0.5)	11.7 (0.6)	0.387
Beans (g)	17.8 (0.3)	16.4 (0.4)	17.1 (0.4)	0.108	19.4 (0.3)	17.3 (0.5)	17.9 (0.7)	0.033
Nuts (g)	0.4 (0.03)	0.5 (0.04)	0.4 (0.03)	0.664	0.33 (0.02)	0.33(0.04)	0.40 (0.05)	0.22
Fruits (g)	111.4 (2.4)	111.3 (3.4)	113.9 (4.0)	0.864	162.1 (3.0)	163.7 (5.0)	154.3 (6.3)	0.355
Vegetables (g)	164.1 (2.0)	151.7 (2.7)	157.2 (3.0)	0.021	162.2 (1.8)	158.9 (3.3)	147.7 (3.5)	0.001
Mushrooms (g)	4.1 (0.1)	3.8 (0.1)	4.2 (0.2)	0.924	4.4 (0.1)	4.0 (0.2)	3. 9(0.2)	0.014
Cakes, cookies and chocolates (g)	4.1 (0.2)	4.2 (0.2)	4.3 (0.2)	0.082	3.9 (0.1)	4.9 (0.3)	6.0 (0.4)	0.0004
Soft drinks (g)	14.0 (0.6)	14.5 (0.7)	17.3 (1.0)	0.001	8.5 (0.5)	9.8 (0.8)	11.5 (1.1)	0.041
Other beverage (g)	35.4 (1.1)	34.6 (1.3)	31.0 (1.4)	0.023	33.0 (1.2)	29.5 (1.7)	27.8 (2.1)	0.008

Values were expressed as means (SE). Food consumption was represented as grams per 1000 kcal. The *p*-trend was obtained in general linear model analysis with adjustment for age, body mass index, alcohol intake, smoking, exercise, education level, and income status.

**Table 3:** Nutritional status of the participants according to the level of liking for fat.

	Men				Women			
	Dislike (*n* = 1895)	Neither Like nor Dislike (*n* = 998)	Like (*n* = 857)	*p*-Trend	Dislike (*n* = 2605)	Neither Like nor Dislike (*n* = 848)	Like (*n* = 528)	*p*-Trend
Total energy intake (kcal)	1947.8 (11.5)	2000.7 (17.3)	2111.5 (18.8)	<0.0001	1771.4 (9.9)	1829.6 (18.1)	1943.6 (24.0)	<0.0001
Nutrient intake								
Carbohydrate (g)	334.3 (0.6)	329.9 (0.9)	326.5 (1.1)	<0.0001	341.2 (0.6)	336.5 (1.1)	336.6 (1.3)	0.018
Protein (g)	64.4 (0.2)	64.5 (0.3)	65.5 (0.4)	0.008	64.1 (0.2)	64.3 (0.4)	63.6 (0.4)	0.076
Fat (g)	31 (0.2)	32.9 (0.3)	33.7 (0.3)	<0.0001	28.9 (0.2)	31.2 (0.4)	30.8 (0.5)	0.005
Cholesterol (g)	165.7 (2.2)	166 (3.0)	173.4 (3.1)	0.029	168.6 (2.3)	178.2 (4.2)	176.8 (5.0)	0.315
Vitamin C (g)	113.5 (1.3)	107.9 (1.7)	108 (1.9)	0.007	134.6 (1.5)	132.7 (2.6)	123.8 (2.9)	0.002
Fiber (mg)	6.7 (0.05)	6.3 (0.1)	6.4 (0.1)	0.0003	7.3 (0.05)	7.0 (0.1)	6.9 (0.1)	<0.0001
Energy distribution								
Carbohydrate (% of total energy)	70.5 (0.1)	69.4 (0.2)	68.2 (0.2)	<0.0001	72.7 (0.1)	71.5 (0.2)	71.0 (0.3)	<0.0001
Protein (% of total energy)	13.6 (0.1)	13.6 (0.1)	13.9 (0.1)	<0.0001	13.3 (0.1)	13.4 (0.1)	13.4 (0.1)	0.699
Fat (% of total energy)	14.8 (0.1)	15.8 (0.2)	16.7 (0.2)	<0.0001	13.0 (0.1)	14.2 (0.2)	14.6 (0.2)	<0.0001

Values were expressed as means (SE). Nutrients were adjusted for total energy intake using residual method. The *p*-trend was obtained in general linear model analysis with adjustment for age, body mass index, alcohol intake, smoking, exercise, education level, and income status.

**Table 4:** Clinical characteristics of the participants according to the level of liking for fat.

	Men				Women			
	Dislike (*n* = 1895)	Neither Like nor Dislike (*n* = 998)	Like (*n* = 857)	*p*-Trend	Dislike (*n* = 2605)	Neither Like nor Dislike (*n* = 848)	Like (*n* = 528)	*p*-Trend
Total cholesterol (mg/dL)	197.8 (0.8)	200.7 (1.1)	202.9 (1.2)	0.019	199.7 (0.7)	196.5 (1.2)	204.8 (1.6)	0.004
HDL cholesterol (mg/dL)	47.5 (0.3)	47.7 (0.4)	47.8 (0.4)	0.005	50.7 (0.2)	51.9 (0.4)	51.3 (0.5)	0.075
LDL cholesterol (mg/dL)	118.4 (0.8)	121.3 (1.1)	122.6 (1.2)	0.01	122.0 (0.6)	118.9 (1.1)	127.0 (1.4)	0.001
Triglycerides (mg/dL)	169.4 (2.9)	168.0 (3.9)	175.3 (4.3)	0.321	137.7 (1.8)	131.2 (2.9)	137.8 (3.9)	0.544
Glucose (mg/dL)	95.2 (0.6)	95.3 (0.7)	95.2 (0.8)	0.409	90.2 (0.4)	90.2 (0.8)	92.7 (1.3)	0.085
Insulin (μIU/mL)	6.9 (0.1)	7.2 (0.1)	7.3 (0.2)	0.858	7.8 (0.1)	8.1 (0.2)	8.5 (0.2)	0.027
HOMA-IR (mg/dL)	1.65 (0.02)	1.72 (0.04)	1.74 (0.04)	0.839	1.75 (0.02)	1.83 (0.04)	1.97 (0.06)	0.005
Systolic blood pressure (mmHg)	116.6 (0.4)	116.5 (0.5)	117.2 (0.5)	0.673	117.3(0.4)	114.6 (0.7)	117.3 (0.9)	0.097
Diastolic blood pressure (mmHg)	75.9 (0.3)	76.3 (0.4)	75.9 (0.4)	0.896	73.7 (0.2)	73.1 (0.4)	73.9 (0.6)	0.121
Waist circumference (cm)	82.7 (0.2)	84.1 (0.2)	85.1 (0.3)	<0.0001	81.0 (0.2)	80.8 (0.3)	82.8 (0.4)	<0.0001
Body mass index (kg/m^2^)	24.0 (0.1)	24.4 (0.1)	24.9 (0.1)	<0.0001	24.8 (0.1)	24.8 (0.1)	25.6 (0.1)	<0.0001

HDL, high-density lipoprotein; LDL, low-density lipoprotein; HOMA-IR, homeostasis model assessment of insulin resistance. Values were expressed as means (SE). The *p*-trend was obtained in general linear model analysis with adjustment for age, alcohol intake, smoking, exercise, education level, and income status.

**Table 5:** Odds ratios (95% confidence intervals) for obesity and metabolic syndrome according to level of liking for fat.

	Men				Women			
	Dislike (*n* = 1895)	Neither Like nor Dislike (*n* = 998)	Like (*n* = 857)	*p*-Trend	Dislike (*n* = 2605)	Neither Like nor Dislike (*n* = 848)	Like (*n* = 528)	*p*-Trend
**Low HDL cholesterol**								
Prevalence (n (%))	427 (24.9)	238 (23.9)	225 (26.3)		1342 (51.5)	396 (46.7)	270 (51.1)	
ORs (95% CI)	1	0.86 (0.71–1.04)	0.94 (0.77–1.14)	0.346 *	1	0.85 (0.72–1.00)	0.92 (0.76–1.11)	0.135 *
**Hypertriglyceridemia**								
Prevalence (n (%))	811 (42.8)	447 (44.8)	404 (47.1)		796 (30.6)	236 (27.8)	166 (31.4)	
ORs (95% CI)	1	0.98 (0.83–1.15)	0.97 (0.82–1.15)	0.715 *	1	1.03 (0.86–1.24)	0.98 (0.79–1.21)	0.956 *
**High fasting glucose**								
Prevalence (n (%))	411 (21.7)	215 (21.5)	178 (20.8)		336 (12.9)	103 (12.2)	77 (14.6)	
ORs (95% CI)	1	0.95 (0.79–1.16)	0.81 (0.66–0.99)	0.048 *	1	1.04 (0.81–1.32)	1.07 (0.81–1.41)	0.609 *
**High blood pressure**								
Prevalence (n (%))	606 (32.0)	305 (30.6)	289 (33.7)		855 (32.8)	226 (26.7)	172 (32.6)	
ORs (95% CI)	1	0.96 (0.81–1.14)	0.90 (0.75–1.08)	0.275 *	1	1.00 (0.82–1.22)	1.03 (0.83–1.29)	0.799 *
**Abdominal obesity**								
Prevalence (n (%))	337 (17.8)	234 (23.5)	251 (29.3)		1386 (53.2)	450 (53.1)	318 (60.2)	
ORs (95% CI)	1	1.45 (1.20–1.75)	1.91 (1.58–2.31)	<0.0001 ^†^	1	1.31 (1.11–1.55)	1.58 (1.29–1.94)	<0.0001 ^†^
**Obesity**								
Prevalence (n (%))	676 (35.7)	426 (42.7)	428 (49.9)		1132 (43.5)	364 (42.9)	294 (55.7)	
ORs (95% CI)	1	1.30 (1.11–1.52)	1.87 (1.58–2.21)	<0.0001 ^†^	1	1.06 (0.91–1.25)	1.72 (1.42–2.08)	<0.0001 ^†^
**Severe obesity**								
Prevalence (n (%))	41 (2.2)	33 (3.3)	33 (3.9)		171 (6.6)	55 (6.5)	50 (9.5)	
ORs (95% CI)	1	1.48 (0.93–2.36)	1.82 (1.14–2.90)	0.01 ^†^	1	1.04 (0.75–1.42)	1.52 (1.09–2.12)	0.027 ^†^
**Metabolic syndrome **(No. of components)								
**≥3**								
Prevalence (n (%))	357 (18.8)	204 (20.4)	199 (23.2)		804 (30.9)	232 (27.4)	176 (33.3)	
ORs (95% CI)	1	1.14 (0.94–1.38)	1.29 (1.06–1.50)	0.01 ^†^	1	1.10 (0.92–1.32)	1.28 (1.04–1.58)	0.018 ^†^
**1–2**								
Prevalence (n (%))	998 (52.7)	521 (52.2)	442 (51.6)		1288 (49.4)	414 (48.8)	264 (50.0)	
ORs (95% CI)	1	0.98 (0.84–1.15)	0.95 (0.81–1.12)	0.566 ^†^	1	0.93 (0.79–1.08)	1.01 (0.84–1.22)	0.772 ^†^
**0 (normal)**								
Prevalence (n (%))	540 (28.5)	273 (27.4)	216 (25.2)		513 (19.7)	202 (23.8)	88 (16.7)	
ORs (95% CI)	1	0.93 (0.78–1.10)	0.86 (0.71–1.03)	0.092 ^†^	1	0.98 (0.81–1.20)	0.70 (0.54–0.91)	0.022 ^†^

CI, confidence intervals; HDL, high density lipoprotein; OR, odds ratio. ORs (95% CIs) were conducted by logistic regression model. * *p*-trend obtained with adjustment for age, body mass index, alcohol intake, smoking, exercise, education level, and income status. ^†^ *p*-trend obtained with adjustment for age, alcohol intake, smoking, exercise, education level, and income status.

## Supplementary Materials

The following are available online at http://www.mdpi.com/2072-6643/10/7/877/s1, Table S1: Un-adjusted odds ratios (95% confidence intervals) for obesity and metabolic syndrome according to level of liking for fat.
