# Supplementary material for: Association of Sensory Liking for Fat with Dietary Intake and Metabolic Syndrome in Korean Adults
Source: Nutrients. 2018 Jul 6;10(7):877. doi: 10.3390/nu10070877 (PMC6073860; doi:10.3390/nu10070877)
Supplement: Supplementary file 1 [file nutrients-10-00877-s001.pdf]

Table S1. Un-adjusted odds ratios (95% confidence intervals) for obesity and metabolic syndrome according to level of liking for fat.

	Men				Women			
	**Dislike** (*n* = 1,895)	**Neither like nor dislike** (*n* = 998)	**Like** (*n* = 857)	***p*-trend**	**Dislike** (*n* = 2,605)	**Neither like nor dislike** (*n* = 848)	**Like** (*n* = 528)	***p*-trend**
Low HDL cholesterol	1	0.94 (0.79 - 1.13)	1.07 (0.89 - 1.29)	0.581	1	0.83 (0.71 - 0.96)	0.99 (0.82 - 1.19)	0.282
Hypertriglyceridemia	1	1.08 (0.93 - 1.27)	1.19 (1.01 - 1.40)	0.032	1	0.88 (0.74 - 1.04)	1.04 (0.85 - 1.28)	0.811
High fasting glucose	1	0.99 (0.82 - 1.19)	0.95 (0.78 - 1.15)	0.609	1	0.93 (0.74 - 1.18)	1.15 (0.88 - 1.51)	0.502
High blood pressure	1	0.94 (0.79 - 1.11)	1.08 (0.91 - 1.29)	0.512	1	0.74 (0.63 - 0.88)	0.99 (0.81 - 1.21)	0.164
Abdominal obesity	1	1.42 (1.17 - 1.71)	1.92 (1.59 - 2.31)	<0.0001	1	0.99 (0.85 - 1.16)	1.33 (1.10 - 1.61)	0.013
Obesity	1	1.34 (1.15 - 1.57)	1.80 (1.53 - 2.12)	0.01	1	0.98 (0.84 - 1.14)	1.64 (1.35 - 1.97)	0.046
Severe obesity	1	1.55 (0.97 - 2.46)	1.81 (1.14 - 2.89)	<0.0001	1	0.99 (0.72 - 1.35)	1.49 (1.09 - 2.07)	<0.0001
**Metabolic syndrome** (No. of components)								
≥ 3	1	1.11 (0.91-1.34)	1.30 (1.07-1.59)	0.009	1	0.84 (0.71-1.00)	1.12 (0.92-1.37)	0.835
1 - 2	1	0.98 (1.84-1.15)	0.96 (0.82-1.13)	0.595	1	0.98 (0.84-1.14)	1.02 (0.85-1.23)	0.940
0 (normal)	1	0.95 (0.80-1.12)	0.85 (0.70-1.02)	0.077	1	1.28 (1.06-1.54)	0.82 (0.64-1.05)	0.740

HDL, high density lipoprotein. Odd ratios (95% confidence intervals) were conducted by logistic regression model.
